# Changes in Endometriosis-Associated Symptoms Following Immunization against SARS-CoV-2: A Cross-Sectional Study

**DOI:** 10.3390/jcm13051459

**Published:** 2024-03-02

**Authors:** Stefan Lukac, Thomas W. P. Friedl, Tobias Gruber, Marinus Schmid, Elena Leinert, Wolfgang Janni, Katharina Hancke, Davut Dayan

**Affiliations:** Department of Obstetrics and Gynecology, University Hospital Ulm, Prittwitzstrasse 43, 89075 Ulm, Germany

**Keywords:** SARS-CoV-2, immunization, COVID-19, dysmenorrhea, dyspareunia, dyschezia, bloating, endometriosis

## Abstract

**Background**: There are many reports about variations in the menstrual cycle after infection with SARS-CoV-2 or vaccination against it. However, data on SARS-CoV-2 infection or vaccination-related changes in menstruation-associated endometriosis-typical symptoms such as dysmenorrhea, dyspareunia, dyschezia, dysuria, and bloating are rare or missing. **Methods**: This retrospective study was performed as an online survey among employees and students at the University Hospital Ulm, Germany. Changes regarding the presence of mentioned symptoms and after immunization (vaccination and/or infection) were evaluated with the McNemar Test. Additionally, the risk factors associated with these changes and associations between a subjectively perceived general change in menstruation and changes in the symptoms were evaluated. **Results**: A total of 1589 respondents were included in the final analysis. Less than 4% of respondents reported the occurrence of new symptoms that they had not experienced before immunization. Overall, there was a significant reduction in the presence of dysmenorrhea, back pain, dyschezia, bloating, and dyspareunia after immunization against coronavirus (*p* < 0.001). Only 2.3% of all participants reported to have been diagnosed with endometriosis. Factors associated with changes in endometriosis-typical symptoms following immunization were body mass index, age, endometriosis, and thyroid disease. **Conclusions**: Our results provide unique data about a reduction in the incidence of endometriosis-associated symptoms as dysmenorrhea, dyschezia, and dyspareunia after immunization against COVID-19.

## 1. Introduction

Since the beginning of the pandemic of coronavirus disease 2019 (COVID-19), there have been emerging reports about disturbances in different medical conditions [1,2,3,4]. The menstrual cycle is one of the most observed parameters regarding women’s health, and therefore, there is a robust amount of data about its alteration after immunization against COVID-19 [3,4,5,6]. These publications describe more intense and prolonged menstrual bleeding after infection or vaccination against COVID-19. Fear of augmented menstruation could potentially lead to avoidance of vaccination, especially among women suffering from severe menstrual symptoms as dysmenorrhea, dyschezia, dysuria, dyspareunia, back pain, or bloating [7]. These are common in endometriosis, but can occur in other medical conditions as well and affect a high proportion of menstruating women [8]. Knowledge about the COVID-19-related dynamics in mentioned symptoms is limited. There are a few studies reporting the effect of immunization against COVID-19 (vaccination or infection) on symptoms in endometriosis patients, focusing mostly on dysmenorrhea [9]. Information from the general population and especially about other conditions is lacking. The goal of our work is to evaluate the dynamics of the incidence of endometriosis-associated symptoms after immunization against COVID-19.

## 2. Materials and Methods

### 2.1. Study Population

Our cross-sectional questionnaire-based study was performed during 1–31 March 2021. A total of 6383 female/diverse employees and students at Ulm University Hospital were requested to fill in an anonymous online survey after providing informed consent. This relatively homogenous group of women of reproductive age represents a collective with a high rate of vaccination and/or infection with COVID-19. In order to increase the participation rate and reduce negative bias through reports of only affected respondents, there was a possibility to win one of three EUR 50 vouchers in a strictly separate lottery. Included were premenopausal women older than 18 years with menstruation who were immunized against COVID-19 and who answered both questions about symptoms before and after immunization. Excluded were respondents who were postmenopausal, responded only to the questions before or only after immunization, and had an unknown immunization status. Immunization includes at least one vaccination against COVID-19 and/or infection with SARS-CoV-2 proven by polymerase-chain-reaction test.

### 2.2. Analyzed Parameters

The specific online questionnaire was designed to target general information such as age, height, weight, and gender. Their body mass index was than calculated as weight/height^2^. Additionally, medical conditions affecting menstruation such as endometriosis, night work, thyroid disease, age at menarche, polycystic ovarian syndrome (PCOS), and the count of pregnancies were recorded. Regarding immunization against COVID-19, different parameters were collected, such as vaccination status (yes/no), infection status (yes/no), immunization status (homogeneous vs. heterogenous), type of vaccine, and combination of vaccines (homogenous vs. heterogenous). To evaluate the changes in the five defined parameters, the participants were asked about having the following symptoms prior to and after immunization. The evaluated symptoms were as follows: painful menstruation (dysmenorrhea), painful defecation (dyschezia), painful urination (dysuria), painful sexual intercourse (dyspareunia), bloating, and backpain during menstrual bleeding. The intensity of the symptoms was not reflected. Finally, every participant was also asked about subjectively perceived general change regarding their menstrual cycle (yes/no) after immunization.

### 2.3. Statistical Analysis

In the descriptive statistics, the continuous variables including age, age at menarche, and BMI are described by median and range. For categorical variables, the absolute and relative frequencies are provided. The McNemar test was used to compare the changes before and after immunization.

Binary logistic regressions were performed to evaluate factors associated with the changes in the observed symptoms. Based on the number of events, we decided to evaluate the non-reporting of the symptoms after immunization (improvement) as the dependent variable. Analyzed factors were age (years), BMI (kg/m^2^), subgroup (employee vs. student), working night shifts (yes vs. no), thyroid dysfunction (yes vs. no), PCOS (yes vs. no), endometriosis (yes vs. no), previous pregnancies (yes vs. no), age at menarche (years), immunization (vaccination with or without infection), and combination of vaccines (heterogenic vs. homogenous). Odds ratios with 95% confidence intervals were calculated for univariable and multivariable binary logistic regressions (including all factors listed above).

Cohen’s kappa coefficient was calculated to assess the agreement between changes in defined symptoms and subjectively perceived changes (yes/no) in the menstrual cycle; the level of agreement was categorized according to Landis and Koch [10].

Statistical analysis was performed with IBM SPSS Statistics software package version 28 (IBM Corp., Armonk, NY, USA) and *p* values less than 0.05 were considered statistically significant.

## 3. Results

A total of 1788 persons completed our survey. A total of 199 were excluded because of being postmenopausal, not vaccinated or infected, and not completing both questions regarding menstrual symptoms before and after immunization. So, 1589 respondents were included in the final analysis of this study. A description of the study population can be found in the Table 1, whereby only 37 (2.3%) of all participants reported to have known endometriosis.

**Figure 1:**
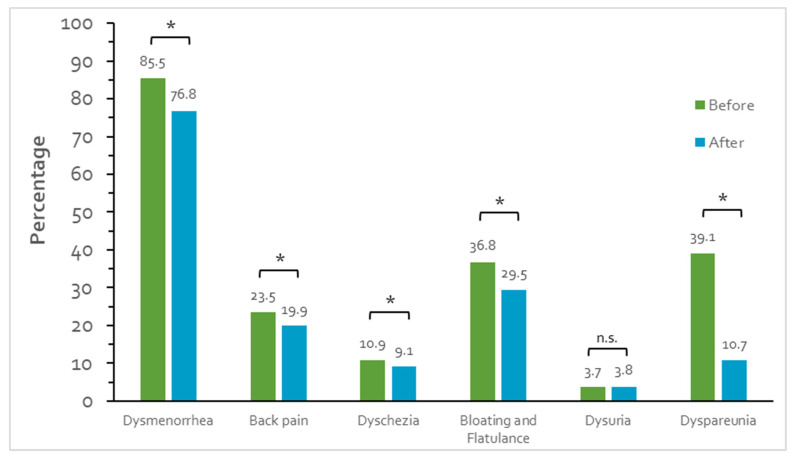
Incidence of menstrual symptoms before and after immunization against COVID-19; *n* = 1589; * *p* < 0.001; n.s.: not significant.

There was a significant reduction in dysmenorrhea [1358 (85.5%) vs. 1220 (76.8%); *p* < 0.001], back pain [373 (23.5%) vs. 316 (19.9%); *p* < 0.001], dyschezia [173 (10.9%) vs. 144 (9.1%); *p* < 0.001], bloating [583 (36.8%) vs. 468 (29.5%) *p* < 0.001], and dyspareunia [621 (39.1%) vs. 170 (10.7%); *p* < 0.001] after immunization against coronavirus, but not for dysuria (3.7% vs. 3.8%; *p* = 0.711) (Figure 1). For each symptom, only a minority reported a new incidence after immunization and the dynamics of these changes can be found in Table 2.

The results of the univariate regression are summarized in the Table 3. As there was no significant change in dysuria, it was not included in the univariable and multivariable regression. In the multivariable analysis adjusted for the factors from the univariate analysis, there was no parameter significantly affecting dysmenorrhea. Back pain was significantly affected by BMI (OR 1.05; 95% CI 1.01–1.10; *p* = 0.010) and thyroid disease (OR 0.34; 95% CI 0.13–1.89; *p* = 0.028). Dyschezia was affected by BMI (OR 1.07; 95% CI 1.01–1.13; *p* = 0.024) and endometriosis (OR 0.19; 95% CI 0.06–0.58; *p* = 0.004). There was tendency for bloating to be affected by BMI (OR 1.04; 95% CI 1.00–1.07; *p* = 0.05). Dyspareunia was affected only by thyroid disease (OR 0.63; 0.41–0.98; *p* = 0.038).

The correlation between perceived changes in the menstrual cycle in general and changes in particular symptoms was not significant for any observed parameter according to Landis and Koch (Table 4).

## 4. Discussion

Our study is the first German study comparing the incidence of endometriosis-associated symptoms such as dysmenorrhea, dyspareunia, dyschezia, dysuria, bloating, and back pain before and after immunization against COVID-19. In our cohort consisting of medical students and employees of a university hospital, there was a high incidence of participants reporting painful menstruation before and after immunization, but only 2.3% of our study population had been diagnosed with endometriosis. This relation is similar to the one reported in the study of Muhaidat [11] and to the incidence of dysmenorrhea in the general population described by previous studies [12,13,14]. Surprisingly, the reported prevalence of dysmenorrhea decreased after immunization by 8.7%. In the previously published literature, Mattar reports a higher incidence of pelvic pain in an immunized cohort in comparison to a non-vaccinated cohort, but does not report the incidence in the same immunized cohort before immunization [15]. Another study from Israel has reported the worsening of dysmenorrhea in the cohort with and without endometriosis, but the information about positive improvement are is [16]. Additionally, another study has reported new incidence or worsening of dysmenorrhea by more than 47% of participants during the COVID-19 pandemic [17]. In contrast, there are also studies reporting reduced occurrence or improvement in their symptoms following COVID-19 vaccination, similar to our results [11,18]. However, the studies focusing on dysmenorrhea or back pain described above are very heterogenous regarding their methods; a few evaluated the intensity of the symptoms [16,17], others compared endometriosis patients vs. health controls [16,19], and only two of these studies followed the same approach that we used here and compared the occurrence of the symptoms before and after immunization [11,18]. The majority of our participants reported dysmenorrhea and back pain less frequently after immunization, but rarely, we also observed the reverse pattern, i.e., dysmenorrhea and back pain after but not prior to immunization, similar to our results regarding changes in menstrual bleeding reported elsewhere [5]. In accordance with previous studies, a high BMI was a protective factor and thyroid disease was a negative risk factor for menstrual back pain after immunization [20,21]. Comparable data regarding risk factors for emerging back pain after immunization against COVID-19 are rare. One study excluded patients with thyroid disease, others do not report on BMI or thyroid dysfunctions [16,17,18,19], and one study found no impact of BMI and thyroid disease on changes regarding dysmenorrhea and back pain following immunization against COVID-19 [11].

With respect to gastrointestinal symptoms, the dominant symptom in our cohort was perimenstrual bloating, while dyschezia was less frequent than perimenstrual bloating prior to immunization. Both symptoms were reported significantly less often after immunization against COVID-19, with a high BMI being positively and the presence of diagnosed endometriosis being negatively associated with the disappearance of dyschezia following immunization, in accordance with previously published studies [22]. The last of the organ-related symptoms, dysuria, was the rarest of the evaluated symptoms and not affected by immunization against COVID-19 in our population. On the contrary, the incidence of dyspareunia dropped by 28.4% following immunization, representing the largest change in an endometriosis-associated symptom observed in our study. Published studies have reported contradictory results, with some studies showing no significant change in dyspareunia before and after vaccination [16,23], while a meta-analysis by Kabani et al. described an increase in dyspareunia during the COVID-19 pandemic in endometriosis patients [9].

Another study showed a decreased pain score in women with penetration disorders during the pandemic [24], which might be explained by less frequent sexual intercourse during the pandemic [25]. The role of thyroid diseases as a risk factor for dyspareunia was previously described [21], confirming our result of a negative effect of thyroid diseases on the likelihood of dyspareunia disappearing following immunization against COVID-19. Finally, subjectively perceived general changes (yes/no) in the menstrual cycle following immunization were not associated with reported changes regarding any of the investigated menstrual symptoms, as shown by the very low Kappa values describing the agreement between perceived general change in the menstrual cycle and changes in each of the symptoms obtained in our study. Obviously, the reported changes in menstrual pain symptoms did not translate into subjectively perceived general changes regarding the menstrual cycle, similar to the lack of association between disturbances in menstrual bleeding and subjectively perceived general changes regarding the menstrual cycle reported previously [5].

The possible causes for the observed decrease in the prevalence of menstrual-related pain symptoms following immunization against COVID-19 are most probably multifactorial, comprising both social consequences of pandemic-related restrictions and immunological factors. Firstly, a general reduction in workload tempo during the pandemic, increased working from home office, reduced social interactions, and a reduction in sexual intercourse could be factors reducing possibly stress-related menstrual pain symptoms [26]. Additionally, symptoms such as dysmenorrhea, dyschezia, and bloating as well as dysuria are mainly hormonally triggered, with estrogen, progesterone, and prostaglandins (PG) playing key roles [27]. PGF2 alpha is considered a key player leading to dysmenorrhea, but PGE works in a contradictory manner, causing relaxation and vasodilatation in myometrium [28]. Previous studies have reported variations in estrogen levels during COVID-19 infection [3] and it is also known that a COVID-19 infection is associated with increased levels of PGE2 [29]. Perhaps the boost in immune systems through immunization against COVID-19 results in some balancing of prostaglandin effects, leading to the reduction in menstrual pain symptoms. Additionally, in comparison to the other cohorts, the women in ours were significantly younger (25 years vs. 29–34) [11,16,17]. The expression of endometrial receptors related to SARS-CoV-2 infection varies during the menstrual cycle, but susceptibility increases with the age of women, and this could be an additional factor leading to discrepancies between our cohorts [9,30]. However, at this point, this is purely speculative and much more research is needed to fully understand the complex interactions between vaccination, hormones, and menstrual symptoms.

Taken together, the currently available published studies provide very heterogeneous results regarding changes in menstrual symptoms following immunization against COVID-19, partly due to different methodology and the use of surrogate parameters [17,19,23,24,25]. Several studies evaluated symptoms only in patients with endometriosis, but not in the general population [9,16,19]. Other studies evaluated only the worsening of the symptoms, the overall effect of pandemics, or just only vaccines, but reports about the incidence of menstrual symptoms in the general population after different immunization methods are not available [6,16,17,23,24]. This heterogeneity of the available data hampers the comparability and thus the interpretation of our results in light of published findings. Another limiting factor is the retrospective data collection, and with it, the risk of not reporting the presence of symptoms before and/or after immunization due to fading memory. Furthermore, our cohort consists of people with significantly higher medical education compared to the general population, which could lead to underreporting if the symptoms were not serious. The strengths of our study, which to our knowledge is the first study reporting such data obtained in Germany, are the large size of our cohort and the inclusion of premenopausal women comprising different age categories. Furthermore, the availability of information on demographic data and comorbidities allowed us to evaluate which factors affected changes in menstrual symptoms in adjusted multivariable analyses.

In conclusion, we could show that the majority of the women in our cohort reported endometriosis-typical symptoms such as dysmenorrhea, back pain, dyschezia, bloating, and dyspareunia less often after compared to before immunization against COVID-19. The mentioned social and immuno-inflammatory factors might be considered to explain these changes, but the etiology seems to be multifactorial. The high incidence of endometriosis-typical pain symptoms in the evaluated population mostly without endometriosis warrants further strategies to approach and reduce painful menstrual symptoms.

## Figures and Tables

**Table 1:** Description of the cohort.

Total		1589 (100%)
**Age (years)**	Median and range	25 (18–59)
**Age at menarche (years)**	Median and range	13 (9–17)
**Body mass index (kg/m^2^)**	Median and range	22.0 (15.7–65.3)
**Subgroup**	Student	907 (57.1%)
	Employee	682 (42.9%)
**Night work**	Yes	297 (18.7%)
	No	1292 (81.3%)
**Thyroid disease**	Hypothyreosis	145 (9.1%)
	Hyperthyreosis	10 (0.6%)
	No	1380 (86.8%)
	Missing	54 (3.4%)
**Polycystic ovarian syndrome**	Yes	74 (4.7%)
	No	1515 (95.3%)
**Endometriosis**	Yes	37 (2.3%)
	No	1552 (97.7%)
**Pregnancies**	0	1347 (84.8%)
	1 or more	242 (15.2%)
**Immunization**	Only vaccination	1130 (71.1%)
	Vaccination and infection	439 (27.6%)
	Missing	20 (1.3%)
**Vaccination**	Homogenous (same vaccine)	1010 (63.6%)
	Heterogenous (different vaccines)	556 (35.0%)
	Missing	23 (1.4)

**Table 2:** Dynamics of the changes in menstrual symptoms after immunization against COVID-19 (*n* = 1589).

	New Symptom	Unchanged	Loss of Symptom
Dysmenorrhea	23 (1.4%)	1373 (86.5%)	193 (12.1%)
Back Pain	12 (0.8%)	1467 (92.3%)	110 (6.9%)
Dyspareunia *	7 (0.4%)	1014 (63.8%)	458 (28.8%)
Dyschezia	12 (0.8%)	1535 (96.6%)	42 (2.6%)
Dysuria	16 (1%)	1560 (98.2%)	13 (0.8%)
Bloating	60 (3.8%)	1353 (85.1%)	176 (11.1%)

* 110 Virgo intacta.

**Table 3:** Results from univariable logistic regression models for improvement (i.e., disappearance yes vs no) in dysmenorrhea, back pain, dyspareunia, dyschezia, and bloating after immunization against COVID-19. OR: odds ratio, CI: confidence interval, *p*: significance. PCOS: polycystic ovarian syndrome.

	Dysmenorrhea		Back Pain		Dyspareunia		Dyschezia		Bloating	
	OR (95% CI)	*p*	OR (95% CI)	*p*	OR (95% CI)	*p*	OR (95% CI)	*p*	OR (95% CI)	*p*
Age (years)	1.01 (0.99–1.03)	0.462	1.00 (0.99–1.03)	0.494	0.98 (0.96–0.99)	0.007	0.99 (0.95–1.03)	0.693	1.01 (0.99–1.03)	0.166
BMI (kg/m^2^)	1.01 (0.98–1.05)	0.454	1.04 (1.01–1.07)	0.035	0.96 (0.94–0.99)	0.02	1.05 (1.01–1.10)	0.029	1.04 (1.01–1.07)	0.021
Subgroup (employee vs. student)	1.05 (0.78–1.43)	0.737	1.09 (0.74–1.60)	0.665	0.72 (0.58–0.90)	0.004	0.64 (0.33–1.23)	0.178	1.21 (0.88–1.66)	0.229
Night work (yes vs. no)	1.00 (0.68–1.47)	0.988	0.66 (0.42–1.04)	0.071	1.15 (0.86–1.52)	0.348	1.71 (0.67–4.34)	0.263	0.88 (0.60–1.30)	0.525
Thyroid disease (yes vs. no)	0.81 (0.47–1.40)	0.456	0.49 (0.21–1.13)	0.094	0.53 (0.35–0.81)	0.003	0.69 (0.21–2.26)	0.537	1.09 (0.65–1.84)	0.736
PCOS (yes vs. no)	0.88 (0.44–1.74)	0.712	1.05 (0.41–2.65)	0.921	0.65 (0.40–1.06)	0.082	2.06 (0.28–15.17)	0.479	0.90 (0.44–1.83)	0.761
Endometriosis (yes vs. no)	0.88 (0.34–2.29)	0.797	0.61 (0.21–1.75)	0.355	0.59 (0.30–1.14)	0.115	0.20 (0.07–0.61)	0.004	0.52 (0.23–1.21)	0.130
Pregnancies (yes vs. no)	1.03 (0.68–1.56)	0.897	1.39 (0.86–2.24)	0.178	0.62 (0.44–0.86)	0.004	1.06 (0.46–2.40)	0.898	1.27 (0.84–1.92)	0.249
Age at menarche (years)	0.96 (0.86–1.06)	0.405	0.98 (0.86–1.13)	0.811	0.96 (0.89–1.04)	0.294	0.89 (0.71–1.10)	0.282	1.01 (0.90–1.12)	0.932
Immunization (infection and vaccination vs. vaccination only)	1.01 (0.72–1.41)	0.98	0.91 (0.60–1.39)	0.670	0.88 (0.69–1.13)	0.884	1.97 (0.87–4.46)	0.106	0.92 (0.65–1.29)	0.623
Vaccination (heterogenous vs. homogenous)	0.82 (0.59–1.13)	0.228	0.70 (0.45–1.07)	0.096	0.82 (0.65–1.03)	0.085	0.81 (0.42–1.57)	0.529	1.07 (0.77–1.48)	0.675

**Table 4:** Agreement between subjectively perceived general changes in the menstrual cycle and changes regarding the presence of particular symptoms after COVID-19 immunization.

Correlation with	Dysmenorrhea	Back Pain	Dyspareunia	Dyschezia	Bloating
Cohen’s Kappa	−0.003	0.001	−0.10	0.002	0.001
*p*	0.492	0.751	0.079	0.218	0.734

## Data Availability

Data is contained within the article.
